# Microwave-Assisted Simultaneous Extraction of Luteolin and Apigenin from Tree Peony Pod and Evaluation of Its Antioxidant Activity

**DOI:** 10.1155/2014/506971

**Published:** 2014-10-22

**Authors:** Hongzheng Wang, Lei Yang, Yuangang Zu, Xiuhua Zhao

**Affiliations:** ^1^Key Laboratory of Forest Plant Ecology, Ministry of Education, Northeast Forestry University, Box 332, Hexing Road 26, Harbin, Heilongjiang 150040, China; ^2^State Engineering Laboratory for Bioresource Eco-Utilization, Northeast Forestry University, Harbin 150040, China

## Abstract

An efficient microwave-assisted extraction (MAE) technique was employed in simultaneous extraction of luteolin and apigenin from tree peony pod. The MAE procedure was optimized using response surface methodology (RSM) and compared with other conventional extraction techniques of macerate extraction (ME) and heat reflux extraction (HRE). The optimal conditions of MAE were as follows: employing 70% ethanol volume fraction as solvent, soaking time of 4 h, liquid-solid ratio of 10 (mL/g), microwave irradiation power of 265 W, microwave irradiation time of 9.6 min, and 3 extraction cycles. Under the optimal conditions, 151 *μ*g/g luteolin and 104 *μ*g/g apigenin were extracted from the tree peony pod. Compared with ME and HRE, MAE gave the highest extraction efficiency. The antioxidant activities of the extracts obtained by MAE, ME, and HRE were evaluated using a 2,2-di(4-*tert*-octylphenyl)-1-picrylhydrazyl (DPPH) free radical-scavenging assay, a ferric reducing antioxidant power assay (FRAP), and a reducing power assay. Meanwhile, the structural changes of the unprocessed and processed tree peony pod samples were analyzed by scanning electron microscopy.

## 1. Introduction

Tree peony was loved as an ornamental for its beautiful flower. During its cultivation for thousands of years, many cultivars of tree peony have been bred. Root cortex of tree peony is also used as an important traditional Chinese medicine (TCM), having been recorded in the Pharmacopoeia of the People's Republic of China [1], which is beneficial for the treatment of diseases related mainly to irregular menstruation and dysmenorrhea [2]. Peony seed oil is rich in *α*-linolenic acid [3], which has beneficial effects on human nutrition and health. The seed oils of Fengdan (*Paeonia ostii*) and Ziban (*Paeonia rockii*) have been approved as new resource food by government of China in 2011. The market demand for peony tree seed oil is growing with the enhancing cognition of its nutrition functions. The remaining pod is around 60% of the total bulk, and it has been either disposed of as landfill waste or used as low-value fuel. A growing attention to the comprehensive utilization of tree peony resource is paid by people. The pod of tree peony contains valuable bioflavonoids. Among the flavonoids, luteolin and apigenin are the main flavones with better pharmacological activities [4].

**Figure 1:**
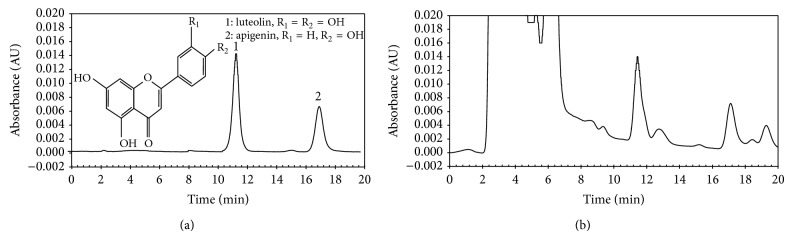
The HPLC profiles of a mixture of standards of luteolin and apigenin (a) and the two compounds in an extract obtained by MAE using 70% ethanol as extraction solvent (b).

Flavonoids are a group of benzo-*γ*-pyran derivatives, comprising a very large class of low molecular weight polyphenol compounds. Among the flavonoids, luteolin (3′,4′,5,7-tetrahydroxyflavone) and apigenin (4′,5,7,-trihydroxyflavone) (Figure 1) are reportedly important functional components, which exhibit the pharmacological effects. For example, luteolin has been found to possess antioxidant [5], anticancer action [6], anti-inflammatory [7], antihepatotoxic action [8], antiallergic, antiosteoporotic [9], antidiabetic [10], and antiplatelet and vasodilatory activity [11]. In addition to antioxidant, anticancer, and anti-inflammatory, apigenin also exhibits antihyperglycemic action [12] and antinociceptive effect [13]. Luteolin and apigenin are natural food additives used extensively in the food and pharmaceutical industries. Flavonoids constitute a large part of global nutraceuticals market [14] and the current nutraceuticals market was estimated at $151 billion in 2011 and was growing by about 6.5% per annum [15]. The pod of tree peony, after flavonoids extraction, could still be used as a high-polysaccharide stock feed in dry form, increasing the potential return for the seed oil industry and reducing the pollution load on the environment.

As far as we know, luteolin and apigenin can be obtained from plant materials, such as* Sesbania grandifolra *[16],* Cajanus cajan *[17],* Apium graveolens *[18],* Platycodon grandiflorum *[19],* Mentha spicata *[20], and* Perilla frutescens *[21]. A new source of luteolin and apigenin would be provided if an efficient extraction technology of luteolin and apigenin from tree peony pod was developed. Meanwhile, it also could be an economical utilization of the disused pod.

Many methods have been developed for separation of luteolin or apigenin from plant materials, such as maceration extraction (ME) [19], Soxhlet extraction [20], and heat reflux extraction (HRE) [21]. However, these extraction techniques are inefficient, time-consuming, and energy-consuming [22]. In recent years, the development and use of environmentally friendly methods have become increasingly popular. Microwave-assisted extraction (MAE) is an extraction technique that offers high reproducibility, short extraction time, simple manipulation, and low solvent consumption, temperature, and energy input [23–26]. MAE utilizes the energy of microwaves to cause dipole rotation of molecules. In the process of MAE, the solvent is rapidly heating and the cell wall of the plant material is quickly destroyed, accelerating the dissolution and extraction of components.

In this paper, the objective is to develop an effective and environment-friendly microwave-assisted approach for the extraction of luteolin and apigenin from the tree peony pod. The influences of the conditions on yields of the luteolin and apigenin were optimized using response surface methodology (RSM). Moreover, the antioxidant activities of the extracts, luteolin, and apigenin were evaluated by DPPH, FRAP, and reducing power assays. Furthermore, the microstructure changes of tree peony pod samples before and after extraction were characterized by scanning electron microscopy (SEM). The present study offers an alternative method for the highly effective utilization of a side product of tree peony utilization.

## 2. Experimental

### 2.1. Plant Materials and Chemicals

Fresh ripe fruits of the cultivated tree peony (*Paeonia ostii*) were hand-harvested in September from Heze (Shandong, China). The pod was separated, cleaned, and dried in oven at 45°C. The dried sample was pulverized using plant grinder (FZ102, Taisite, Tianjin, China) and then sieved (80–120 mesh) before use. Apigenin (≥95%), luteolin (≥97%), gallic acid (≥97.5%), rutin (≥94%), Folin-Ciocalteu reagent, 2,2-di(4-*tert*-octylphenyl)-1-picrylhydrazyl (DPPH, 95%), 6-hydroxy-2,5,7,8-tetramethylchroman-2-carboxylic acid (Trolox, 97%), and 2,4,6-tris(2-pyridyl)-*s*-triazine (TPTZ, ≥98%) were purchased from Sigma-Aldrich Co. LLC (St. Louis, MO, USA). Ethanol, sodium nitrite (NaNO_2_), aluminum trichloride (AlCl_3_) and sodium hydroxide (NaOH), sodium carbonate (Na_2_CO_3_), acetic acid, hydrochloric acid (HCl), ferric chloride (FeCl_3_), sodium dihydrogen phosphate (NaH_2_PO_4_), disodium hydrogen phosphate (Na_2_HPO_4_), potassium ferricyanide, and trichloroacetic acid (TCA) were purchased from Sinopharm Chemical Reagent Co., Ltd. (Beijing, China). Methanol of chromatographic grade (99.9%) was purchased from J&K Scientific Ltd. (Beijing, China). All solvents and chemicals except methanol were of analytical grade. Reverse osmosis Milli-Q water (Millipore, Bedford, MA, USA) was used for all solutions and dilutions. All of the solvents prepared were filtered through 0.45 *μ*m microporous membrane (Guangfu, Tianjin, China).

### 2.2. Apparatus

The experimental setup of the microwave extraction apparatus was from Wang et al. [23] and Liu et al. [24]. Briefly, a domestic WP700 microwave-assisted extraction unit (Glanz, Guangdong, China) with a 2450 MHz magnetron and power continuously adjustable was used in the extraction step. The dimensions of the interior cavity of the oven are 215 mm × 350 mm × 330 mm. It was modified in our laboratory with the addition of a water condenser whose wall was coated with polytetrafluoroethylene to prevent the leakage of microwaves. A round-bottom flask with a capacity of 100 mL was placed in the oven and connected to a reflux condenser. The whole system was run at atmospheric pressure and could be employed at the maximum power of 700 W.

### 2.3. MAE Procedure

1.00 g of the ground dried pod sample was mixed with ethanol solution in a 100 mL round bottom flask. Soaked for a certain time, then the suspension was extracted by MAE. The optimum ethanol volume fraction, soaking time, liquid-solid ratio, microwave irradiation power, microwave irradiation time, and number of extraction cycles were systematically studied in this work. After MAE, it was cooled to room temperature rapidly by a cold bath and centrifuged with 10,000 ×g for 10 minutes. The supernatant was collected for subsequent HPLC analysis.

### 2.4. Optimization MAE by Response Surface Method (RSM)

In order to highlight the most influential factors and possible interactions, the operating conditions were optimized by RSM using the Box-Behnken software in data processing. Box-Behnken design was applied using Design-Expert 8.06 without any blocking. The bounds of the factors were 6–10 ratio of liquid-solid, 120–385 W of microwave irradiation power, and 6–10 minutes of microwave irradiation time. The specific protocols for the experiments were shown in Table 1.

### 2.5. Traditional Reference Extraction Procedure

The ME and HRE experiments were operated under the optimized conditions. 1.00 g of the ground dried pod sample was mixed with 10 mL 70% volume fraction of ethanol in a 100 mL round bottom flask. The main technical parameters used were listed in Table 2. After completion of extraction, the liquid retentate was decanted and filtered through Whatman number 2 filter paper (Whatman International Limited, Kent, England). The filtrate was concentrated in a vacuum evaporator (R206, Senco Technology Co. Ltd., Shanghai, China) at 60°C and then lyophilized in a freeze-dryer (Scientz-10N, Ningbo Scientz Biotechnology Co., Ltd, China) to obtain crude extract. The cold trap temperature was −56°C and the vacuum was less than 1 Pa. The crude extract was collected and stored in 4°C until it was used.

### 2.6. HPLC Analysis and Quantification

The HPLC system consisted of a Waters 1525 Binary HPLC Pump, 2489 UV/Visible Detector, and automatic column temperature control box. Chromatographic separation was performed on Aichrom Bond-AQ C18 reversed-phase column (4.6 mm × 250 mm, 5 *μ*m, Abel Industries, Canada).

For HPLC analysis, the mobile phase was methanol-water-phosphate acid (30 : 69.3 : 0.7, v/v/v), and the flow rate was 1 mL/min. The column temperature was maintained at 25°C. The wavelength used for luteolin and apigenin was 360 nm. 10 *μ*L example was injected and the run time was 20 min. The retention times for luteolin and apigenin were 11.4 and 17.1 min (Figure 1), respectively. Under these conditions, the two flavonoids were baseline separated. Luteolin and apigenin were identified by comparing their retention time with corresponding peaks in the standard solution.

Corresponding calibration curves for luteolin and apigenin were *Y*_luteolin_ = 63937*X* + 37891 (*R* ^2^ = 0.9999) and *Y*_apigenin_ = 73716*X* + 24357 (*R* ^2^ = 0.9998). A good linearity was found for each of luteolin and apigenin in the range of 1–150 and 0.67–85 *μ*g/mL, respectively.

### 2.7. Determination of Total Flavonoids

The total flavonoids contents of crude extracts obtained by MAE, ME, and HRE were determined by the method of Guo et al. [27] with some modifications. Sample (0.16 mg/mL; 2.5 mL) was mixed with 0.15 mL 5% NaNO_2_ solution. After 6 min, 0.15 mL 10% AlCl_3_ was added. After another 6 min, 2 mL 4% NaOH was added. The absorbance was measured at 510 nm using a UV-Vis spectrophotometer (Shimadzu UV-2550; Shimadzu, Kyoto, Japan) after incubation for 15 min. The determination was performed in triplicate. Quantification was done on the basis of the standard curve of rutin.

### 2.8. Determination of Total Phenolics

The total phenolics contents of crude extracts obtained by MAE, ME, and HRE were determined by Folin-Ciocalteu method [28] with little modifications. Briefly, 0.5 mL sample (0.12 mg/mL) was mixed with 2.8 mL H_2_O and 0.5 mL Folin-Ciocalteu reagent. After 3 min, 1.5 mL 7.5% Na_2_CO_3_ was added. The reaction mixture was mixed thoroughly and incubated at 40°C for 30 min in the dark. Absorbance was then measured at 765 nm using the spectrophotometer (UV-2550, Shimadzu, Japan). Gallic acid was used to calculate the standard curve (0.06–0.3 mg/mL). The determination was performed in triplicate.

### 2.9. Evaluation of Antioxidant Capacity

To evaluate antioxidant capacities of crude extracts obtained by MAE, ME, and HRE, their DPPH radical-scavenging activity, FRAR, and reducing power were determined, with standard compounds of luteolin and apigenin as the positive controls.

DPPH radical-scavenging activity was measured according to the method of Zu et al. [29] with a slight modification. 0.1 mL of each sample at different concentration (0.25–1.6 mg/mL in 70% ethanol) was added to 3.9 mL 25 mg/mL DPPH solution in 95% ethanol. The mixture was mixed vigorously and allowed to stand at room temperature in the dark for 30 min. Then the absorbance was measured at 517 nm, with absolute ethanol as the control, and the DPPH radical-scavenging activity was calculated.

FRAP was assayed according to Liu et al. method [30]. Briefly, a working solution was prepared freshly by mixing 250 mL sodium acetate buffer (pH 3.6, 300 mM), 25 mL TPTZ solution (10 mM, in 40 mM HCl), and 25 mL 20 mM FeCl_3_ solution. The mixture was incubated at 37°C for 30 min and was referred to as FRAP solution. 0.15 mL of each sample at different concentration (0.05–0.25 mg/mL in 70% ethanol) was mixed with 2.85 mL FRAP solution and allowed standing in the dark for 30 min. Then the absorbance at 593 nm was measured. A standard curve was prepared using Trolox ranging from 37.5 to 600 *μ*M. The FRAP was expressed as *μ*mol Trolox equivalents (TE)/g crude or positive control.

Reducing power assay was following the method of Zu et al. [29]. 0.5 mL of each sample at different concentration (0.015–0.25 mg/mL in 70% ethanol) was mixed with 1.5 mL sodium phosphate buffer (pH 6.6, 0.2 M) and 1.5 mL 1% potassium ferricyanide solution. The mixture was incubated in a water bath at 50°C for 20 min. Then, 1.5 mL 10% trichloroacetic acid (TCA) solution and 3 mL water was added. The mixture was mixed vigorously and the absorbance was measured at 707 nm. Increased absorbance of the mixture indicated greater reducing power.

### 2.10. SEM Observation

In order to investigate the effects of MAE, ME, and HRE on morphological alterations of the pod material, the pod material and residues of MAE, ME, and HRE were scanned with a SEM system (Quanta-200, FEI Company, USA). Samples were fixed on a specimen holder with aluminium tape and then sputtered with the gold and examined under high vacuum condition at an accelerating voltage of 10 kV (20 *μ*m, 1000x magnification).

### 2.11. Statistical Analysis

Statistical analyses were performed with SPSS Statistics (Version 19, IBM Company, USA). All experimental results were the average of three parallel measurements expressed as the mean ± standard deviation (SD). The Fisher test value (*F*-value) was obtained from the ANOVA test generated by the software.* P* values < 0.05 were regarded as significant.

## 3. Results and Discussion

### 3.1. Optimization of Luteolin and Apigenin Extraction Using a Factorial Design

The univariate method was used to optimize the following parameters: ethanol volume fraction in the extraction solvent, pod soaking time, liquid-solid ratio, microwave irradiation power, microwave irradiation time, and extraction cycles.

#### 3.1.1. Effect of Ethanol Volume Fraction

Species and concentration of solvent is regarded as one of the most important parameters for MAE, which affects the solubility of the target component and the absorption of microwave energy [31, 32]. Although methanol has a great advantage in MAE for its best absorbance of the microwave energy, it is not recommended in food processing because of its toxicity [33]. So mixture of ethanol and water is recommended. In this study, the extractions were carried out with aqueous ethanol solutions at different concentrations (ethanol volume fraction from 0 to 90%) and with other conditions of 1 h soaking time, 10 (mL/g) of liquid-solid ratio, 385 W of microwave irradiation power, 10 min of microwave irradiation time, and one extraction cycle.

**Figure 2:**
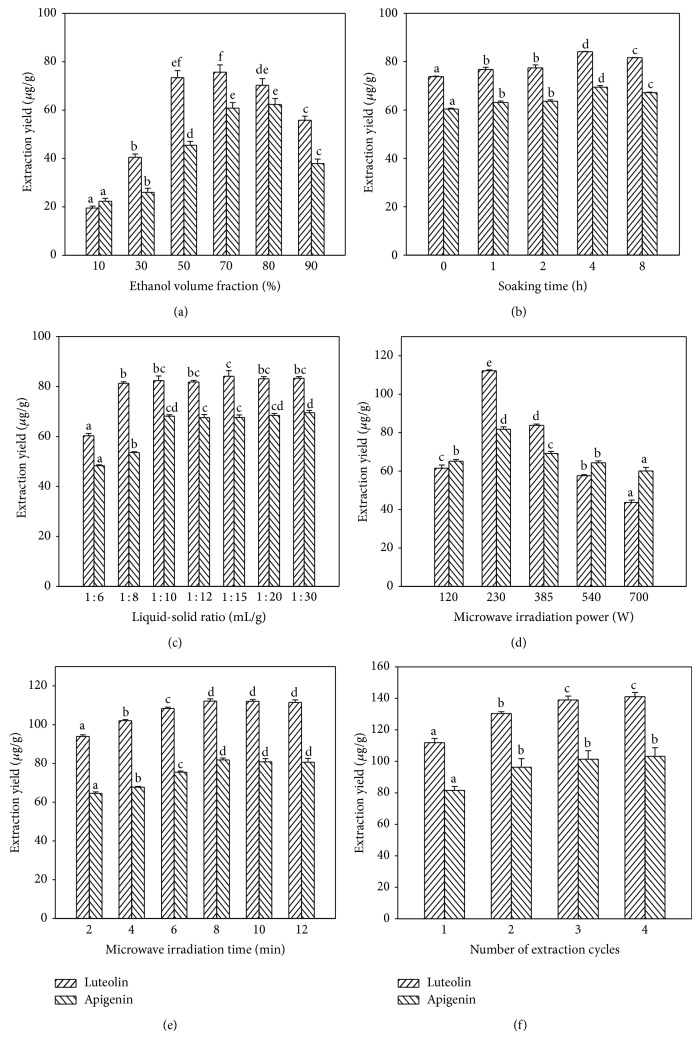
The influence conditions ((a): ethanol volume fraction; (b): soaking time; (c): liquid-solid ratio; (d): microwave irradiation power; (e): microwave irradiation time; (f): number of extraction cycles) on the extraction yields of luteolin and apigenin. The values represent means ± standard deviation. Values followed by the same letter in the same assay are not significantly different (*P* > 0.05, *n* = 3).

Ethanol volume fraction significantly affected the extraction yields of luteolin and apigenin (Figure 2(a)). The extraction yield of luteolin significantly increased with the increase of ethanol volume fraction ranging from 0 to 50% and then decreased when ethanol volume fraction was higher than 80%. It was found that the higher ethanol volume fraction was needed to extract apigenin than luteolin from the matrix, which was likely due to the smaller polarity of apigenin. The most efficient ethanol volume fractions for luteolin and apigenin were 50% and 70%, respectively. Taking into account both of the total extraction yields and the economization on ethanol, 70% was considered for the optimal ethanol volume fraction.

#### 3.1.2. Effect of Pod Soaking Time

Using of the technique of soaking can get better extraction efficiency of target compound in microwave-assisted processing [34]. Experiments were conducted to investigate the effect of soaking time on yields of luteolin and apigenin in MAE. Pod samples were soaked in 70% ethanol for 0, 1, 2, 4, or 8 h. Then they were extracted in the microwave oven for 10 min at 385 W. Information of the effect of soaking time on the extractions of luteolin and apigenin was given in Figure 2(b). A substantial increase in the extraction yield was obtained after soaking the pod. The target ingredients extraction yields increased significantly when the soaking time was 0–4 h (*P* < 0.05); however longer soaking time did not lead to further increases in yield. Therefore, 4 h was chosen as the optimal soaking time.

#### 3.1.3. Effect of Liquid-Solid Ratio

In an extraction process, it is important to maximize extraction yield but also to minimize the consumption of solvent. Inadequate solvent leads to low operational efficiency and excessive solvent leads to waste. For investigating the influence of liquid-solid ratio on extraction yields of luteolin and apigenin, tests were performed at different liquid-solid ratios ranging from 6 to 30 (mL/g). The results showed that increasing liquid-solid ratio enhanced the extraction yields of luteolin and apigenin (Figure 2(c)), which was due to the increasing of driving force generated from the gradient concentration [35]. In the tested ratio ranging from 10 to 30, no significant difference was found in extraction yields of luteolin and apigenin (*P* > 0.05), which was due to the excessive swelling of the materials caused by the large volume of solvent, according to the reports of Yan et al. [33] and Ma et al. [36]. Hence, a value of 10 (mL/g) was considered the optimal liquid-solid ratio for the MAE process.

#### 3.1.4. Effect of Microwave Irradiation Power

Microwave irradiation power is the other important factor affecting extraction yield of component in MAE. A higher microwave power increased the temperature of the mixture of the extraction solvent and sample, leading to higher mass transfer rates of substances from the sample [37]. In this study, the effect of irradiation power ranging from 120 to 700 W on extraction yields of luteolin and apigenin was investigated (Figure 2(d)). It was found that the extraction yields of luteolin and apigenin significantly increased when the microwave irradiation power increased from 120 W to 230 W (*P* < 0.05) and gradually decreased with the further increasing of irradiation power, which might be due to either thermal or oxidative degradation of luteolin and apigenin by the excessive irradiation. The results indicated that the optimum extraction condition was the use of the microwave power of 230 W.

#### 3.1.5. Effect of Microwave Irradiation Time

The influence of microwave irradiation time on the extraction yields of the luteolin and apigenin was examined over a range of 2–12 min; other factors were fixed at 70% ethanol volume fraction as extraction solvent, soaking time 4 h, liquid-solid ratio 10 (mL/g), microwave irradiation power 230 W, and one extraction cycle. As shown in Figure 2(e), the extraction yields of luteolin and apigenin sharply increased with the increase of microwave irradiation time in the beginning of MAE. Luteolin and apigenin could reach their optimum extraction yields at 8 min during the extraction process. When microwave irradiation time was above 8 min, no significant variation was found in extraction yields of luteolin and apigenin (*P* > 0.05). Thus, a microwave irradiation time of 8 min was chosen as the optimal microwave irradiation time.

#### 3.1.6. Number of Extraction Cycles

Adequate extraction cycle contributes to the full extraction of interesting substances from the residue. The extraction yields of luteolin and apigenin were investigated in MAE process conducted 1–4 cycles, with other conditions of 70% ethanol volume fraction, 4 h of soaking time, 10 (mL/g) of liquid-solid ratio, 230 W of microwave irradiation power, and 8 min of microwave irradiation time. The 2nd cycle significantly increased the yields of luteolin and apigenin (*P* < 0.05). It was the 3rd extraction cycle, but not the 4th cycle, that significantly enhanced the extraction yield of luteolin of MAE (*P* < 0.05) (Figure 2(f)). For saving solvent, energy, and time, three-cycle extraction was sufficient to extract luteolin and apigenin present in tree peony pod.

### 3.2. Optimization Extraction Conditions by Response Surface Method (RSM)

In order to study the most influential factors and the interactions between the factors, the liquid-solid ratio, microwave irradiation power, and time were optimized by RSM. In Table 1, the total extraction yield was defined as the sum of yields of luteolin and apigenin in an extraction experiment. The surface response analysis for the total extraction yield indicated that the effects of all the three factors on the response were significant with a positive linear relationship (*P* < 0.05). The empirical relationship between the total extraction yield and the extraction parameters was generated as follows:

(1) Y=−397.079+61.705A+1.880B+15.070C+0.007AB +0.250AC−0.006BC−3.125A2−0.004B2−0.875C2.

**Figure 3:**
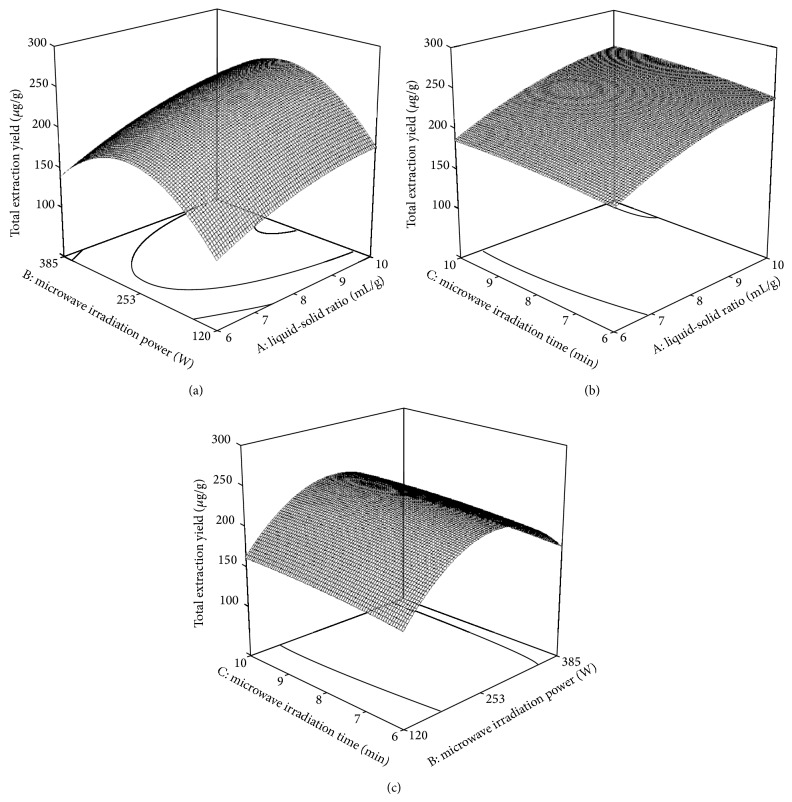
Response surface plots showing the effects of variables on total extraction yield. (a) Interaction of liquid-solid ratio and microwave irradiation power; (b) interaction of liquid-solid ratio and microwave irradiation time; (c) interaction of microwave irradiation power and time.

It was found that there was no significance in the lack of fit (*P* > 0.05). The ANOVA analysis results showed that the quadratic model was valid for the spatial influence of variables on the response. Additionally, the *R* ^2^ value for the model was 0.996, which conformed with the fact that the model could adequately represent the true relationships between the three parameters. In this case, *A*, *B*, *C*, *AB*, *A* ^2^, *B* ^2^, and *C* ^2^ were the significant model terms. To highlight the interactions of the three factors on total extraction yield, 3D profiles of the model were illustrated in Figure 3, when the other parameters were kept constant.

The interaction of liquid-solid ratio and microwave irradiation power was shown in Figure 3(a). In these experiments, the other parameters were kept as follows: 70% ethanol volume fraction, 4 h of soaking time, 8 min of microwave irradiation time, and 3 extraction cycles. Increase of the microwave irradiation power from 120 W to 265.6 W with the liquid-solid ratio increasing from 6 to 10 (mL/g) enhanced the total extraction yield. The further increase of the microwave irradiation power decreased the total extraction yield, likely due to the degradations of luteolin and apigenin under the high irradiation power. Additionally, denser contours were found along the axis of microwave irradiation power than along the axis of liquid-solid ration, indicating that microwave irradiation power had more influence on the total extraction yield than liquid-solid ratio.

Figure 3(b) represented the interaction of liquid-solid ratio and microwave irradiation time. In these experiments, the other parameters were kept as follows: 70% ethanol volume fraction, 4 h of soaking time, 230 W of microwave irradiation power, and 3 extraction cycles. A steady increase of total extraction yield was found when the liquid-solid ratio increased from 6 to 10 (mL/g) along with the increase of the microwave irradiation time from 6 to 9.6 min. A comparison of contour density along axis of liquid-solid ratio and microwave irradiation time suggested that the total extraction yield was more influenced by the liquid-solid ratio than microwave irradiation time.

The response surface for total extraction yield with various microwave irradiation powers and time was showed in Figure 3(c). In this case, the parameters of ethanol volume fraction, soaking time, liquid-solid ratio, and extraction cycles were fixed at 70%, 4 h, 10 (mL/g), and 3, respectively. Being the same as that found in Figure 3(a), the total extraction yield was elevated when the microwave irradiation power increased from 120 to 265.6 W along with the increase of microwave irradiation time from 6 to 9.6 min. The denser contours along the axis of microwave irradiation power indicated that the parameter has a greater impact on the total extraction yield.

From the analysis of RSM, the optimal conditions of MAE can be summarized as follows: 70% ethanol volume fraction, 4 h soaking time, 10 (mL/g) of liquid-solid ratio, 265.6 W of microwave irradiation power, 9.6 min of microwave irradiation time, and 3 extraction cycles. Under these conditions, the total extraction yields can reach 256 *μ*g/g. Taking into account the feasibility in the actual operation, the parameter of microwave irradiation power was adjusted to 265 W. To verify the model, MAE was done three times under these conditions. The actual total extraction yield was 255 *μ*g/g with a deviation of −0.39%.

Under the conditions optimized by RSM, the extraction yields of luteolin and apigenin from tree peony pod were, respectively, 151 *μ*g/g and 104 *μ*g/g. Luteolin and apigenin have also been reported in some other plant materials, such as* S. grandifolra*,* C. cajan*, and* A. graveolens*. The contents of luteolin and apigenin in them were, respectively, in range of about 20–550 *μ*g/g and 40–730 *μ*g/g [16–18]. The data indicated that the extract of tree peony pod would have favorable application value.

### 3.3. Comparison of MAE with Other Methods

The extraction yields of luteolin and apigenin from tree peony pod with MAE, ME, and HRE were compared, and the results were shown in Table 2. The extraction yields of luteolin and apigenin were significantly higher with MAE than with HRE and ME (*P* < 0.05). The total extraction yield of MAE, HRE, and ME was 255, 220, and 145 *μ*g/g, respectively. The time of MAE with energy consumption was also shorter, with the HRE taking 120 min and the MAE only 24 min. The comparison of MAE with ME showed that the microwave irradiation energy was important for increasing the extraction efficiency. MAE was an efficient method for the simultaneous extraction of luteolin and apigenin from tree peony pod.

### 3.4. Evaluation of Antioxidant Activities

#### 3.4.1. DPPH Assay

**Figure 4:**
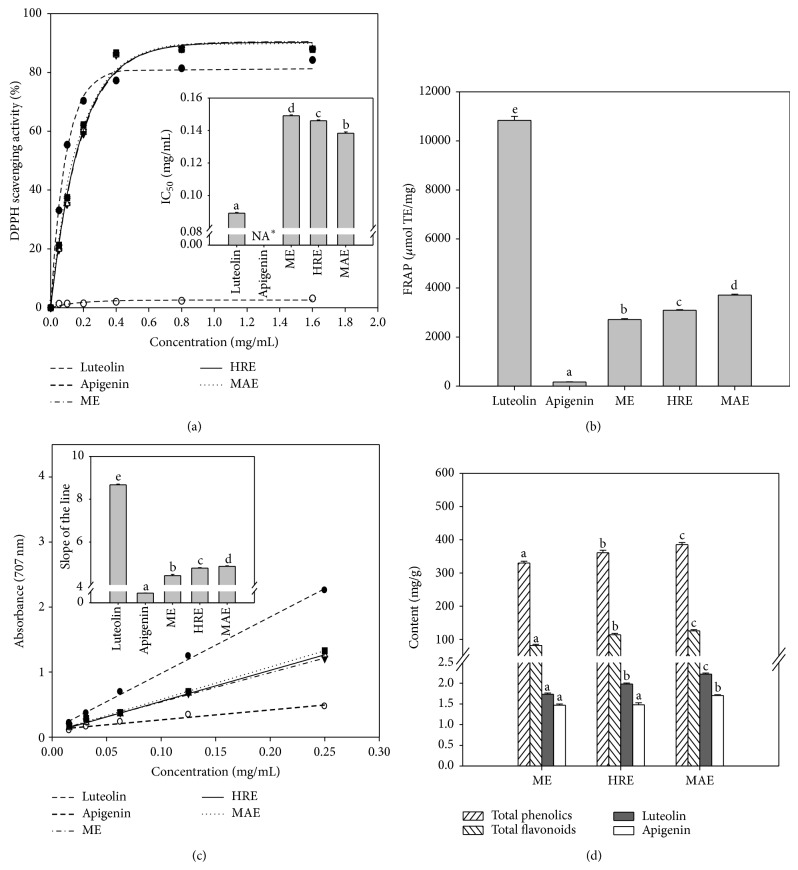
Antioxidant activity and antioxidants contents of crude extracts obtained by ME, HRE, and MAE of tree peony pod. (a) DPPH scavenging activity of the crude extracts; (b) FRAP of the crude extracts; (c) reducing power of the crude extracts; (d) total phenolics, total flavonoids, luteolin, and apigenin contents of the crude extracts. The values represent means ± standard deviation. Values followed by the same letter in the same assay are not significantly different (*P* > 0.05, *n* = 3). Note: ∗ means the value of IC_50_ cannot been obtained.

In the present investigation, the DPPH assay was used to evaluate the radical-scavenging activity of tree peony pod extracts obtained by MAE, ME, and HRE, as well as of pure luteolin and apigenin. As shown in Figure 4(a), all extracts obtained by MAE, HRE, and ME possessed high scavenging activity of DPPH radical. The IC_50_ values of the three extracts were higher than that of the standard compound of luteolin and lower than apigenin. In the three extracts, the highest scavenging activity for DPPH radical was found in the extract obtained by MAE, suggesting that more compounds with hydrogen donating capabilities were extracted in MAE than in ME or HRE.

#### 3.4.2. Ferric Reducing Antioxidant Power Assay

Antioxidant potential of the three extracts obtained by MAE, HRE, and ME was estimated from their abilities to reduce TPTZ-Fe (III) complex to TPTZ-Fe (II) complex, with pure luteolin and apigenin. Among all the samples, luteolin showed the highest FRAP (10838 *μ*mol TE/mg) and apigenin showed the lowest FRAP (163 *μ*mol TE/mg). Similar to the result in DPPH assay, MAE showed the pronounced effect on FRAP. The FRAP of extract obtained by MAE was higher than those obtained by HRE and ME, 20.0 ± 1.4% and 36.9 ± 3.3%, respectively (Figure 4(b)).

#### 3.4.3. Reducing Power Assay

The reducing powers of the extracts obtained by MAE, HRE, and ME were also tested in another system, in which the Fe^3+^/ferricyanide complex transformed into its ferrous form. The amount of Fe^2+^/ferricyanide complex was monitored by measuring the formation of Perl's Prussian blue at 707 nm. Higher absorbance value indicated the higher reducing power [29, 38]. As shown in Figure 4(c), the reducing powers of all samples were concentration dependent ranging from 0.016 mg/mL to 0.25 mg/mL, and good linear relationships were found between the samples concentration and the reducing powers. Agreeing with the DPPH and FRAP tests, the reducing powers of the crude extracts and the pure compounds were arranged as follow: luteolin > MAE > HRE > ME > apigenin.

From the results of DPPH, FRAP, and reducing power assay, it was obvious that MAE was beneficial for the extraction of the antioxidant substances. Polyphenols are the most abundant antioxidants in the plants and provide the main antioxidation in human diet [39]. In the three extracts, the highest contents of total phenolics and total flavonoids were found in the extract obtained by MAE (Figure 4(d)), which was in agreement with the results of antioxidant activities tests* in vitro*. In our study, antioxidant activities of luteolin were obviously higher than apigenin, which was consistent with the results in other studies [40–42]. The contents of luteolin and apigenin were also determined in the three extracts, and a positive relationship was found between the content of luteolin and antioxidative capacity of extract, but not the content of apigenin. It indicated that luteolin was an important antioxidant in the extract.

### 3.5. Structural Changes after Extraction

**Figure 5:**
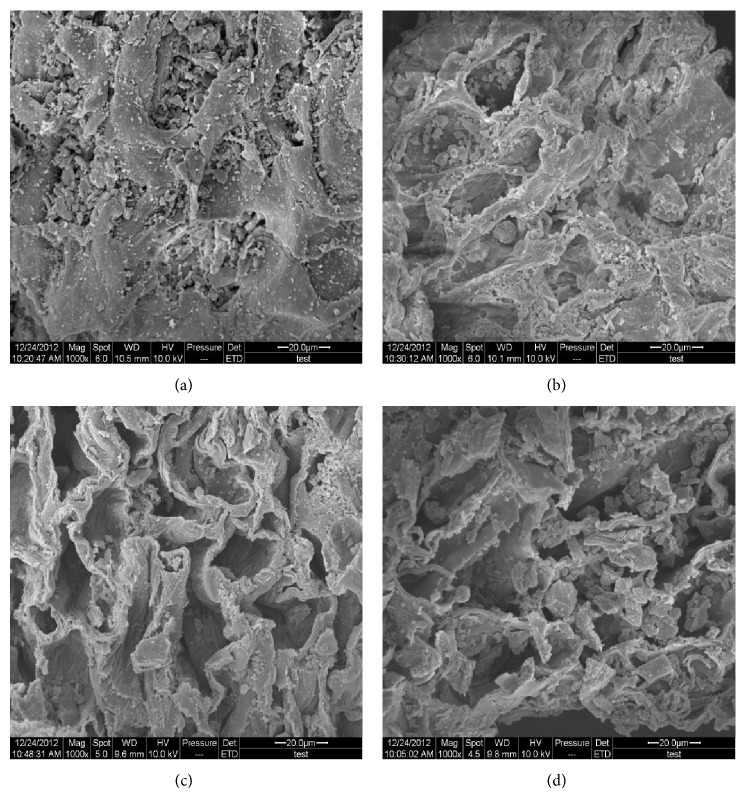
SEM images (20.0 lm, 15.0 kV) of untreated tree peony pod sample (a), sample after ME (b), sample after HRE (c), and sample after MAE (d).

The various extraction methods produced different physical changes in tree peony pod. To investigate these physical changes during MAE, HRE, and ME, the pod samples were analyzed by SEM.  Figure 5 displayed the micrographs of the pod samples before and after the different extraction methods. As shown in Figure 5(a), nubby parenchyma and nondestructed cell walls could be observed in the untreated raw material. No obvious change was found in sample with ME treatment compared to the untreated raw material, except for a few perforations (Figure 5(b)), which might be caused by the dissolution and denaturation of some compositions of cell wall. After HRE or MAE treatment, the sample became a crumbly texture (Figures 5(c) and 5(d)). However, a higher degree of damage was found in the sample with MAE treatment. MAE treatment induced clear wrinkle, dispersion, and fragmentation of external and internal cell walls of the particles, which might be due to the severe thermal stress and localized high pressure [43]. When the sample was subjected to irradiation, rapid heating of polar molecules in material and solvent led to the rapid expansion of the solvent volume, which built up a pressure within the cell. As a result of the continuous increase of the pressure, the structure of cell walls of sample particles was disrupted and that helped the rapid release of substances inside of the material particles to solvents [44].

## 4. Conclusions

In the present study, an efficient MAE method for simultaneous extraction of luteolin and apigenin from tree peony pod has been developed. The optimum conditions for MAE were studied using RSM. Under the optimized conditions, 151 *μ*g/g luteolin and 104 *μ*g/g apigenin were obtained. Compared to other methods, the proposed approach provides higher extraction yields and significantly reduced energy consumption time. Higher antioxidant activities were found in extract obtained by MAE than those obtained by HRE and ME, being estimated in* in vitro* systems of DPPH, TPTZ-Fe (III), and Fe^3+^/ferricyanide complex. This indicated that more antioxidants were extracted from the pod using MAE. In these antioxidants, luteolin might be an important constituent responsible for the high antioxidant activities of the tree peony pod extracts. SEM results showed that MAE disintegrated the rigid wood material efficiently, increasing the release of secondary metabolites. The method may also prove to be useful in the development of efficient and energy saving extraction methods for other flavonoids.

## Figures and Tables

**Table 1:** Experimental design matrix to screen important variables for total extraction yield of luteolin and apigenin.

Run	Factor A Liquid-solid ratio (mL/g)	Factor B Microwave irradiation power (W)	Factor C Microwave irradiation time (min)	Response Total extraction yield (*μ*g/g)
1	8	230	8	228
2	8	120	10	163
3	8	230	8	231
4	8	120	6	150
5	6	230	6	179
6	6	120	8	117
7	10	120	8	175
8	10	230	10	251
9	6	230	10	187
10	8	230	8	231
11	8	385	10	182
12	8	230	8	230
13	8	385	6	175
14	10	230	6	239
15	8	230	8	230
16	6	385	8	138
17	10	385	8	204

**Table 2:** Comparison of MAE with other extraction methods, mean ± S.D (*n* = 3).

Number	Extraction method	Extraction time (h)		Solvent consumption (mL/g)	Extraction yield ± SD (*μ*g/g)		
		Soak time (h)	Energy consumption time (min)		Luteolin	Apigenin	Total
1	ME	36	0	30	82 ± 4^a^	63 ± 3^a^	145
2	HRE	0	120	30	131 ± 6^b^	89 ± 4^b^	220
3	MAE	2	24	30	151 ± 7^c^	104 ± 4^c^	255

^a^Values followed by the same letter in the same assay are not significantly different (*P* > 0.05).

## References

[1] Chinese Pharmacopoeia Commission (2010). *Pharmacopoeia of the People’s Republic of China*.

[2] Wang X., Cheng C., Sun Q., Li F., Liu J., Zheng C. (2005). Isolation and purification of four flavonoid constituents from the flowers of *Paeonia suffruticosa* by high-speed counter-current chromatography. *Journal of Chromatography A*.

[3] Wang C., Zhang P., Dong Y. (2009). The technique of the extraction of oil from peony seed with supercritical CO_2_ extraction method and the analysis of the composition. *Journal of the Chinese Cereals and Oils Association*.

[4] Yi J., Zhu W., Ma H., Wang Y. (2009). Studies on chemical constituents from seeds of *Paeonia suffruticosa* Andr. *Natural product Research and Development*.

[5] Madhesh M., Vaiyapuri M. (2012). Effect of luteolin on lipid peroxidation and antioxidants in acute and chronic periods of isoproterenol induced myocardial infarction in rats. *Journal of Acute Medicine*.

[6] Cai X., Ye T., Liu C., Lu W., Lu M., Zhang J., Wang M., Cao P. (2011). Luteolin induced G2 phase cell cycle arrest and apoptosis on non-small cell lung cancer cells. *Toxicology in Vitro*.

[7] Wu M. J., Weng C. Y., Ding H. Y., Wu P. J. (2005). Anti-inflammatory and antiviral effects of *Glossogyne tenuifolia*. *Life Sciences*.

[8] Chang J., Hsu Y., Kuo P., Kuo Y., Chiang L., Lin C. (2005). Increase of Bax/ Bcl-XL ratio and arrest of cell cycle by luteolin in immortalized human hepatoma cell line. *Life Sciences*.

[9] di Carlo G., Mascolo N., Izzo A. A., Capasso F. (1999). Flavonoids: old and new aspects of a class of natural therapeutic drugs. *Life Sciences*.

[10] Zarzuelo A., Jiménez I., Gámez M. J., Utrilla P., Fernadez I., Torres M. I., Osuna I. (1996). Effects of luteolin 5-O-*β*-rutinoside in streptozotocin-induced diabetic rats. *Life Sciences*.

[11] Chen Y. T., Zheng R. L., Jia Z. J., Ju Y. (1990). Flavonoids as superoxide scavengers and antioxidants. *Free Radical Biology and Medicine*.

[12] Yamagata K., Tagawa C., Matsufuji H., Chino M. (2012). Dietary apigenin regulates high glucose and hypoxic reoxygenation-induced reductions in apelin expression in human endothelial cells. *Journal of Nutritional Biochemistry*.

[13] Pinheiro M. M. G., Boylan F., Fernandes P. D. (2012). Antinociceptive effect of the *Orbignya speciosa* Mart. (Babassu) leaves: evidence for the involvement of apigenin. *Life Sciences*.

[14] Patel J. M. A. (2008). Review of potential health benefits of flavonoids. *Lethbridge Undergraduate Research Journal*.

[15] BBC Research Nutraceuticals : Global Markets and Processing Technologies. http://www.bccresearch.com/market-research/food-and-beverage/nutraceuticals-markets-processing-technologies-fod013d.html.

[16] Miean K. H., Mohamed S. (2001). Flavonoid (myricetin, quercetin, kaempferol, luteolin, and apigenin) content of edible tropical plants. *Journal of Agricultural and Food Chemistry*.

[17] Fu Y.-J., Liu W., Zu Y.-G., Tong M.-H., Li S.-M., Yan M.-M., Efferth T., Luo H. (2008). Enzyme assisted extraction of luteolin and apigenin from pigeonpea [*Cajanuscajan* (L.) Millsp.] leaves. *Food Chemistry*.

[18] Han D., Row K. H. (2011). Determination of luteolin and apigenin in celery using ultrasonic-assisted extraction based on aqueous solution of ionic liquid coupled with HPLC quantification. *Journal of the Science of Food and Agriculture*.

[19] Park S. W., Cho C. S., Ryu N. H., Kim J. H., Kim J. S. (2012). Luteolin extracted from *Platycodon grandiflorum* protects retinal pigment epithelial cells from oxidative stress-induced caspase-3 dependent apoptosis. *Biomedicine and Preventive Nutrition*.

[20] Bimakr M., Rahman R. A., Taip F. S., Ganjloo A., Salleh L. M., Selamat J., Hamid A., Zaidul I. S. M. (2011). Comparison of different extraction methods for the extraction of major bioactive flavonoid compounds from spearmint (*Mentha spicata* L.) leaves. *Food and Bioproducts Processing*.

[21] Zhao G., Qin G. W., Wang J., Chu W. J., Guo L. H. (2010). Functional activation of monoamine transporters by luteolin and apigenin isolated from the fruit of *Perilla frutescens* (L.) Britt. *Neurochemistry International*.

[22] Xiao X., Si X., Tong X., Li G. (2011). Preparation of flavonoids and diarylheptanoid from *Alpinia katsumadai hayata* by microwave-assisted extraction and high-speed counter-current chromatography. *Separation and Purification Technology*.

[23] Wang S.-Y., Yang L., Zu Y.-G. (2011). Design and performance evaluation of ionic-liquids-based microwave-assisted environmentally friendly extraction technique for camptothecin and 10-hydroxycamptothecin from samara of camptotheca acuminata. *Industrial & Engineering Chemistry Research*.

[24] Liu T., Sui X., Zhang R., Yang L., Zu Y., Zhang L., Zhang Y., Zhang Z. (2011). Application of ionic liquids based microwave-assisted simultaneous extraction of carnosic acid, rosmarinic acid and essential oil from *Rosmarinus officinalis*. *Journal of Chromatography A*.

[25] Ma C.-H., Liu T.-T., Yang L., Zu Y.-G., Chen X., Zhang L., Zhang Y., Zhao C. (2011). Ionic liquid-based microwave-assisted extraction of essential oil and biphenyl cyclooctene lignans from *Schisandra chinensis* Baill fruits. *Journal of Chromatography A*.

[26] Ma C.-H., Yang L., Zu Y.-G., Liu T.-T. (2012). Optimization of conditions of solvent-free microwave extraction and study on antioxidant capacity of essential oil from *Schisandra chinensis* (Turcz.) Baill. *Food Chemistry*.

[27] Guo T., Wei L., Sun J., Hou C.-L., Fan L. (2011). Antioxidant activities of extract and fractions from *Tuber indicum* Cooke & Massee. *Food Chemistry*.

[28] Yang L., Huang J.-M., Zu Y.-G., Ma C.-H., Wang H., Sun X.-W., Sun Z. (2011). Preparation and radical scavenging activities of polymeric procyanidins nanoparticles by a supercritical antisolvent (SAS) process. *Food Chemistry*.

[29] Zu S., Yang L., Huang J., Ma C., Wang W., Zhao C., Zu Y. (2012). Micronization of taxifolin by supercritical antisolvent process and evaluation of radical scavenging activity. *International Journal of Molecular Sciences*.

[30] Liu X., Jia J., Yang L., Yang F., Ge H., Zhao C., Zhang L., Zu Y. (2012). Evaluation of antioxidant activities of aqueous extracts and fractionation of different parts of *Elsholtzia ciliata*. *Molecules*.

[31] Yang L., Sun X., Yang F., Zhao C., Zhang L., Zu Y. (2012). Application of ionic liquids in the microwave-assisted extraction of proanthocyanidins from *Larix gmelini* bark. *International Journal of Molecular Sciences*.

[32] Liu Y., Yang L., Zu Y., Zhao C., Zhang L., Zhang Y., Zhang Z., Wang W. (2012). Development of an ionic liquid-based microwave-assisted method for simultaneous extraction and distillation for determination of proanthocyanidins and essential oil in Cortex cinnamomi. *Food Chemistry*.

[33] Yan M.-M., Liu W., Fu Y.-J., Zu Y.-G., Chen C.-Y., Luo M. (2010). Optimisation of the microwave-assisted extraction process for four main astragalosides in *Radix Astragali*. *Food Chemistry*.

[34] Chen F., Mo K., Liu Z., Yang F., Hou K., Li S., Zu Y., Yang L. (2014). Ionic liquid-based vacuum microwave-assisted extraction followed by macroporous resin enrichment for the separation of the three glycosides salicin, hyperin and rutin from Populus bark. *Molecules*.

[35] Chen F., Hou K., Li S., Zu Y., Yang L. (2014). Extraction and chromatographic determination of shikimic acid in Chinese conifer needles with 1-benzyl-3-methylimidazolium bromide ionic liquid aqueous solutions. *Journal of Analytical Methods in Chemistry*.

[36] Ma C., Yang L., Wang W., Yang F., Zhao C., Zu Y. (2012). Extraction of dihydroquercetin from *Larix gmelinii* with ultrasound-assisted and microwave-assisted alternant digestion. *International Journal of Molecular Sciences*.

[37] Yao H., Du X., Yang L., Wang W., Yang F., Zhao C., Meng X., Zhang L., Zu Y. (2012). Microwave-assisted method for simultaneous extraction and hydrolysis for determination of flavonol glycosides in Ginkgo foliage using brönsted acidic ionic-liquid [HO_3_S(CH_2_)_4_mim]HSO_4_ aqueous solutions. *International Journal of Molecular Sciences*.

[38] Liu Z., Ma C., Yang L., Zu Y. (2013). Process optimization of ultrasonic-assisted extraction of arabinogalactan from dihydroquercetin extracted residues by response surface methodology and evaluation of its antioxidant activity. *Journal of Chemistry*.

[39] Lu Q., Liu W., Yang L., Zu Y., Zu B., Zhu M., Zhang Y., Zhang X., Zhang R., Sun Z., Huang J., Li W. (2012). Investigation of the effects of different organosolv pulping methods on antioxidant capacity and extraction efficiency of lignin. *Food Chemistry*.

[40] Brighente I. M. C., Dias M., Verdi L. G., Pizzolatti M. G. (2007). Antioxidant activity and total phenolic content of some Brazilian species. *Pharmaceutical Biology*.

[41] Majewska M., Skrzycki M., Podsiad M., Czeczot H. (2011). Evaluation of antioxidant potential of flavonoids: an *in vitro* study. *Acta Poloniae Pharmaceutica: Drug Research*.

[42] Yi T., Chen Q., He X., So S., Lo Y., Fan L., Xu J., Tang Y., Zhang J., Zhao Z., Chen H. (2013). Chemical quantification and antioxidant assay of four active components in *Ficus hirta* root using UPLC-PAD-MS fingerprinting combined with cluster analysis. *Chemistry Central Journal*.

[43] Golmakani M.-T., Rezaei K. (2008). Comparison of microwave-assisted hydrodistillation withthe traditional hydrodistillation method in the extractionof essential oils from *Thymus vulgaris* L. *Food Chemistry*.

[44] Kong Y., Zu Y.-G., Fu Y.-J., Liu W., Chang F.-R., Li J., Chen Y.-H., Zhang S., Gu C.-B. (2010). Optimization of microwave-assisted extraction of cajaninstilbene acid and pinostrobin from pigeonpea leaves followed by RP-HPLC-DAD determination. *Journal of Food Composition and Analysis*.

